# Dysregulation of Complement Activation and Placental Dysfunction: A Potential Target to Treat Preeclampsia?

**DOI:** 10.3389/fimmu.2019.03098

**Published:** 2020-01-15

**Authors:** E. Pierik, Jelmer R. Prins, Harry van Goor, Gustaaf A. Dekker, Mohamed R. Daha, Marc A. J. Seelen, Sicco A. Scherjon

**Affiliations:** ^1^Department of Obstetrics and Gynecology, University Medical Center Groningen, University of Groningen, Groningen, Netherlands; ^2^Department of Pathology and Medical Biology, University Medical Center Groningen, University of Groningen, Groningen, Netherlands; ^3^Department of Obstetrics and Gynecology, Lyell McEwin Hospital, University of Adelaide, Adelaide, SA, Australia; ^4^Division of Nephrology, Department of Internal Medicine, University Medical Center Groningen and University of Groningen, Groningen, Netherlands

**Keywords:** complement, placental dysfunction, preeclampsia, pregnancy, treatment, review

## Abstract

Preeclampsia is one of the leading causes of maternal and neonatal mortality and morbidity worldwide, affecting 2–8% of all pregnancies. Studies suggest a link between complement activation and preeclampsia. The complement system plays an essential role in the innate immunity, leading to opsonization, inflammation, and elimination of potential pathogens. The complement system also provides a link between innate and adaptive immunity and clearance of immune complexes and apoptotic cells. During pregnancy there is increased activity of the complement system systemically. However, locally at the placenta, complement inhibition is crucial for the maintenance of a normal pregnancy. Inappropriate or excessive activation of the complement system at the placenta is likely involved in placental dysfunction, and is in turn associated with pregnancy complications like preeclampsia. Therefore, modulation of the complement system could be a potential therapeutic target to prevent pregnancy complications such as preeclampsia. This review, based on a systematic literature search, gives an overview of the complement system and its activation locally in the placenta and systemically during healthy pregnancies and during complicated pregnancies, with a focus on preeclampsia. Furthermore, this review describes results of animal and human studies with a focus on the complement system in pregnancy, and the role of the complement system in placental dysfunction. Various clinical and animal studies provide evidence that dysregulation of the complement system is associated with placental dysfunction and therefore with preeclampsia. Several drugs are used for prevention and treatment of preeclampsia in humans and animal models, and some of these drugs work through complement modulation. Therefore, this review further discusses these studies examining pharmaceutical interventions as treatment for preeclampsia. These observations will help direct research to generate new target options for prevention and treatment of preeclampsia, which include direct and indirect modulation of the complement system.

## Introduction

In pregnancy, protection of mother and fetus against potential pathogens is essential, therefore a functional immune system is required (1). Fetal tissues are directly exposed to the maternal blood (2). Consequently, the maternal immune system needs to tolerate these tissues to avoid potential immune attacks at the feto-maternal interface (2, 3). In the placenta, villous tissue covered by fetal trophoblast is in strict connection with the maternal vascular system, where the internal layer is formed by mononucleated cytotrophoblasts and the external layer by multinucleated syncytiotrophoblast (2). Extravillous trophoblast cells also invade the decidua reaching the myometrium and the spiral arteries (2). The endothelial cells of the spiral arteries are partly substituted by endovascular (extravillous) trophoblasts (2). A healthy pregnancy is associated with immunological changes, especially locally at the feto-maternal interface, characterized by the presence of uterine NK cells, and the induction of regulatory T cells both essential for spiral artery remodeling, normal placental development, and preventing fetal tissue rejections (4). The complement system plays an important role in these processes (1). It recruits and activates immune cells which leads to opsonization, inflammation, and lysis of potential pathogens (1, 2). Control of complement activation at the feto-maternal interface is therefore important for a successful pregnancy, and may otherwise lead to adverse pregnancy outcomes including preeclampsia (3).

Preeclampsia is an important cause of maternal and neonatal mortality and morbidity worldwide, affecting 2–8% of all pregnancies (1). It is described by the development of endothelial cell dysfunction, presenting as hypertension, and proteinuria after 20 weeks of gestation (1, 3, 5). Preeclampsia, especially when its onset is early in pregnancy, is associated with placental dysfunction, reduced placental blood flow and fetal growth restriction (FGR) (3). Also, the kidney develops severe damage resulting in renal insufficiency, proteinuria, and abnormal renal histology (5). The immune system is thought to play an important role in the pathogenesis of preeclampsia, including changes in the complement system (1). It is known that activation of the complement system is involved in renal injury in preeclampsia (5). Nowadays, the treatment of preeclampsia is the delivery of the placenta and therefore also of the fetus. Even though delivery is positive for the mother, it may not be beneficial for the fetus, as it might be born preterm (6).

In this review we will discuss various clinical and animal studies providing evidence that dysregulation of the complement system is associated with placental dysfunction and with preeclampsia. Several drugs have been used as prevention and treatment options for preeclampsia in human and animal models, and some of these drugs possibly work through complement modulation. Therefore, this review also discusses these studies examining pharmaceutical interventions as treatment for preeclampsia, with that we hope to help directing research to generate new target options for prevention and treatment of preeclampsia.

## Method

For this review we aimed to provide an overview of the complement system, and its activation locally in the placenta, and systemically during normal healthy pregnancy, and complicated pregnancies, focusing on preeclampsia. Therefore, we undertook a systematic search of the published literature on animal and human studies, which investigated the role of the complement system in placental dysfunction, as it occurs in preeclampsia. A list with articles was generated using combinations of the following search terms in Pubmed: “placental dysfunction, placental insufficiency, placenta, trophoblast, feto-maternal interface, preeclampsia, pre-eclampsia, complement system, complement activation, and complement”. Papers from this list were selected on the basis of quality and relevance. Also, we abstracted information on pharmaceutical interventions of drugs which are currently used for prevention and treatment of preeclampsia in humans and animal models, investigating the role of complement modulation in these treatments of preeclampsia. On the basis of these articles, we created a perspective on the future prospects for targeting the complement system in novel therapeutic strategies for treatment of preeclampsia.

## The Complement System

**Figure 1:**
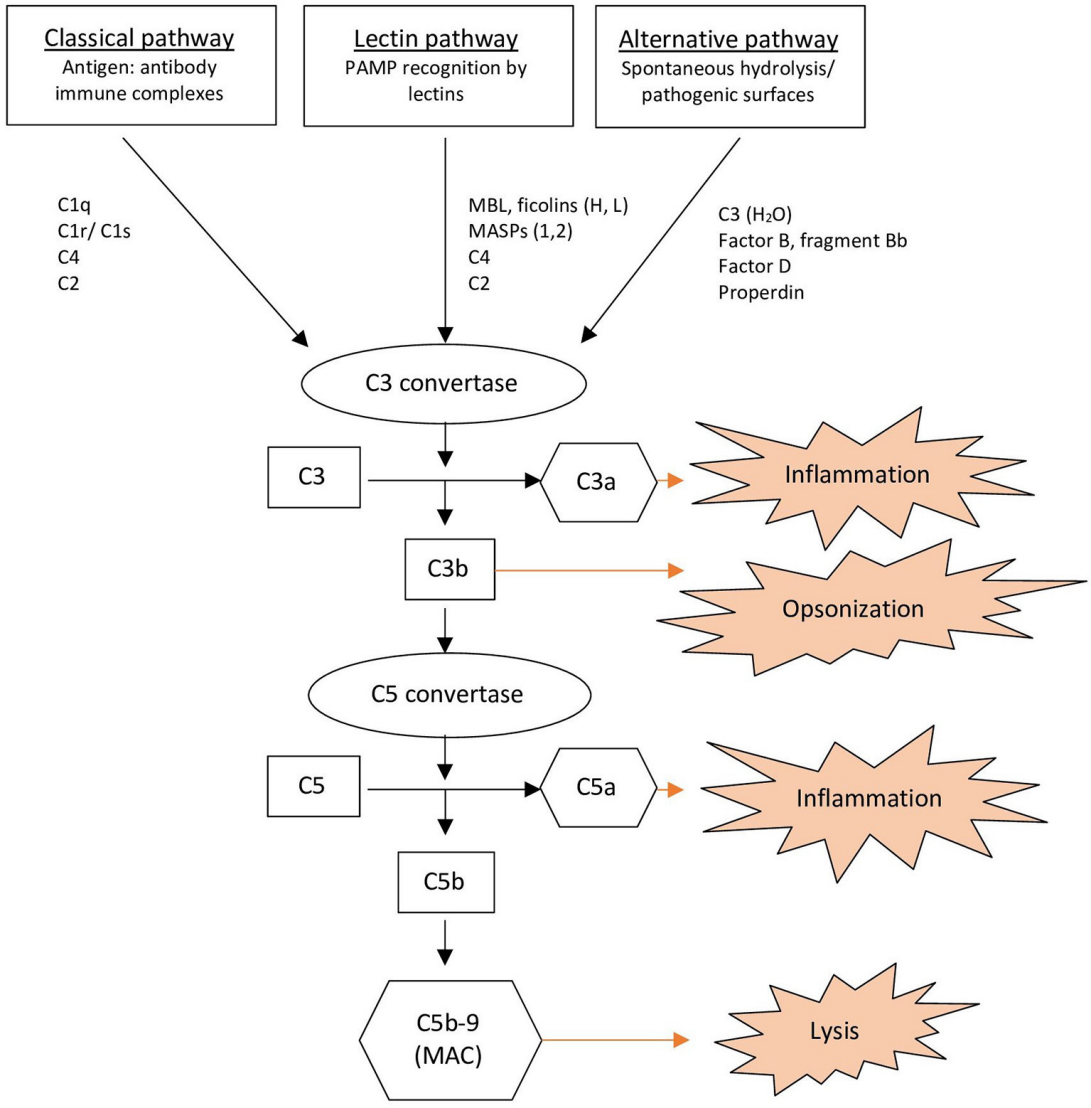
Overview of the complement system. MAC, Membrane Attack Complex; MASPs, Mannose-associated Serine Proteases; MBL, Mannan-binding Lectin; PAMP, Pathogen-associated Molecular Pattern.

The complement system consists of more than 30 proteins present in the plasma and on cell surfaces (1, 7). When the complement system is activated, it induces recruitment and activation of immune cells resulting in opsonization, inflammation and lysis of potential pathogens, immune complexes, and apoptotic cells (Figure 1) (1, 7). The complement system provides a linkage between innate and adaptive immunity (7). Activation of the complement system can be achieved via three pathways: the classical, alternative and lectin pathway (1, 7). All pathways converge to create C3 convertases that split C3 into its active components, C3a, and C3b (1). C3b binds covalently to the activating surface of its target and also contributes to the activation of a C5 convertase. C5 convertase cleaves C5 into C5a and C5b. C5b then associates with C6, C7, C8, and C9 to form the membrane-attack-complex (MAC; C5b-9), which cause lysis (1, 2, 7). Anaphylatoxins, C3a, and C5a, induce chemotaxis and release cytokines (1).

The fetal cells must avoid recognition and activation of the complement system to achieve adequate trophoblast invasion and healthy placentation (3, 8). Normal placentation is achieved by local expression of regulators of the complement system, such as DAF (CD55), CD59, and MCP (CD46), by inhibiting C3, and C5 convertases, and the assembly of MAC on the membrane surface (1–3). Complement components and regulators are present at the placenta to provide immune protection and also to favor implantation and placentation (3). C1q is important in trophoblast migration and spiral artery remodeling, contributing to normal placental development (1). Potential complement activation occurs because of the development of maternal antibodies, recognition of Pathogen-associated Molecular Patterns (PAMPs) or spontaneous activation of the system by exposure to villous structures (7). An excessive increase of complement activation may damage invading extra villous trophoblasts or placental syncytiotrophoblast and is associated with adverse pregnancy outcomes, including preeclampsia (2, 9).

## Complement Activation After Preeclampsia

Several human studies, as seen in Table 1, report an association between placental deposition of complement factors and dysregulation of placental function, particularly preeclampsia. The number of patients in these studies is in general small. Similarly, placental dysfunction is also seen in fetal growth restriction (FGR), but human data on complement dysregulation in this condition is even more scarce. Complement deposits can be the consequence of either excessive activation or inadequate regulation of the complement system (1, 9). Complement activation is thought to be related to inflammatory events in trophoblastic tissue, possibly subsequent to dysregulation of placental angiogenesis (2).

**Table 1:** Overview of complement deposits in human placentas and circulating complement factors of pregnant women with preeclampsia compared to controls.

**Complement pathway**	**Complement factor**	**Human—placenta**	**Human—blood**
Classical	C1q	- Higher deposition at chorionic villi; (10) PE *n* = 20, controls *n* = 20 - Lower deposition at the syncytiotrophoblast; (9) PE = 12, controls *n* = 8 - Higher deposition in controls and EOP compared to LOP in endothelia of placental blood vessels; (9) EOP = 7, LOP *n* = 5, controls *n* = 8 - Comparable, deposition in syncytiotrophoblast and intravillous endothelial cells; (11) PE *n* = 28, controls *n* = 30	- Lower levels; (12) PE *n* = 30, controls *n* = 30
	C4bp	- Higher percentage in controls compared to PE; (9) PE = 12, controls *n* = 8	N.a.
Lectin	C4	N.a.	- Lower levels; (13) PE *n* = 26, controls *n* = 25 (14) PE *n* = 88, controls *n* = 107 (12) PE *n* = 30, controls *n* = 30
	C4d	- Higher deposition in preterm PE with FGR compared to preterm PE without FGR; (15) preterm PE-FGR *n* = 21, preterm PE *n* = 20 - Higher deposition at the syncytiotrophoblast and an association between diffuse deposition in PE placentas and lower gestational age; (11) PE *n* = 28, controls *n* = 30 - Higher deposition, as well in OD as in autologous pregnancies; (16) OD+PE *n* = 9, non-donor PE *n* = 46, OD *n* = 33, IVF *n* = 20	- Higher levels; (17) PE *n* = 60, controls *n* = 60 (18) PE *n* = 60, controls *n* = 60
	Ficolins (H, L)	- High concentration, especially in the syncytiotrophoblast; (19) PE *n* = 5, controls *n* = not specified, statistical analysis performed not specified	- Lower levels; (19) PE *n* = 20, controls *n* = 45 - L-ficolin: lower levels; (18) PE *n* = 60, controls *n* = 60 - H-ficolin: comparable levels; (18) PE *n* = 60, controls *n* = 60
	MBL	- Absent; (11) PE *n* = 28, controls *n* = 30	- Comparable levels; (19) PE *n* = 20, controls *n* = 45 - Higher median levels; (20) PE *n* = 99, controls *n* = 187 - MBL-MASP2: comparable levels; (21) PE *n* = 60, controls *n* = 60
Alternative	C3	- Higher deposition in the decidua of the basal plate and in the villi; (22) PE *n* = 15, controls *n* = 13, no statistical analysis performed - Higher deposition in decidual tissue; (23) PE *n* = 5, controls *n* = 5 - Higher deposition in the endothelial cells of villi; (24) PE *n* = 6, controls *n* = 6 - Higher C3d deposition circumferentially on trophoblast basal membrane; (10) PE *n* = 20, controls *n* = 20 - Comparable, deposition in syncytiotrophoblast layer and villous stroma; (9) EOP *n* = 7, LOP *n* = 5, controls *n* = 10	- Comparable levels; (14) PE *n* = 88, controls *n* = 107 (25) EOP *n* = 30, LOP = 78–80, controls *n* = 94–97
	Fragment Bb	N.a.	- Higher levels; (26) severe PE *n* = 24, controls *n* = 20 - Higher levels in umbilical venous plasma; (26) severe PE *n* = 15, controls *n* = 15 - Early predictor of PE, 3 times more likely to develop PE (27)
Anaphylatoxins	C3a	- Lower C3aR mRNA and protein expression in Hofbauer cells in the villous stroma in severe EOP; (28) severe EOP *n* = 19, controls *n* = 8 - Lower C3aR protein expression in severe PE; (29) severe PE *n* = 52, controls *n* = 66	- Comparable levels; (30) cohort *n* = 668, PE *n* = 31, at 10–15 weeks of gestation - Higher levels; (17) PE *n* = 60, controls *n* = 60 (18) PE *n* = 60, controls *n* = 60 (25) EOP *n* = 30, LOP = 78–80, controls *n* = 94–97 (29) severe PE *n* = 52, controls *n* = 66 - Higher levels in EOP compared to LOP; (25) EOP *n* = 30, LOP = 78–80
	C5a	- Comparable, C5aR detected in syncytiotrophoblast and endothelial cells; (28) severe EOP *n* = 19, controls *n* = 8 - Higher C5aR expression at trophoblast cells; (31) PE *n* = 6, controls *n* = 6 - Lower C5aR protein expression in severe PE; (29) severe PE *n* = 52, controls *n* = 66	- Comparable levels; (12) PE *n* = 30, controls *n* = 30 - Higher levels; (29) severe PE *n* = 52, controls *n* = 66
MAC	C5b-9	- More intense deposition, found in the decidua of the basal plate, in villous stroma and in vessel walls; (22) PE *n* = 15, controls *n* = 13, no statistical analysis performed - Higher C9 deposition, circumferentially distributed; (10) PE *n* = 20, controls *n* = 20 - Higher C9 deposition in severe PE compared to mild PE in villi; (10) severe PE *n* = 10, mild PE *n* = 10 - Higher C9 deposition in villi; (32) PE *n* = 10, control *n* = 11 - Comparable, deposition in basal membrane of syncytiotrophoblast and in villous stroma; (9) EOP *n* = 7, LOP *n* = 5, controls *n* = 10	- Comparable levels; (12) PE *n* = 30, controls *n* = 30 (30) cohort *n* = 668, PE *n* = 31, at 10–15 weeks of gestation - Higher levels; (17) PE *n* = 60, controls *n* = 60 (18) PE *n* = 60, controls *n* = 60 (25) EOP *n* = 30, controls *n* = 94–97 - Higher levels PE-FGR compared to PE without FGR; (17) *n* not specified

*EOP, early-onset preeclampsia; FGR, fetal growth restriction; IVF, in vitro fertilization; LOP, late-onset preeclampsia; n, number; N.a., not available; OD, oocyte donation; PE, preeclampsia*.

Besides complement activation locally in placental tissue, various studies explored complement activation in the circulation of women with preeclampsia. Table 1 gives an overview of several factors which are investigated, with different outcomes. Inconsistency between studies could be the consequence of differences in study populations, including pregnancy duration (early-onset vs. late-onset), ethnicity, sample size, severity of the disease, and method of analyzing circulating levels. Although results are sometimes inconsistent, it is hypothesized that the complement system is involved in preeclampsia (12–14, 17, 18).

### Classical Pathway

#### Classical Pathway—Placenta

Modulation of the classical complement pathway in women with preeclampsia was already observed in 1984. Higher deposition of C1q at chorionic villi was observed in preeclamptic placental tissues compared to normal pregnancy placental tissues (10). At the syncytiotrophoblast layer, Lokki et al. overall observed less C1q in preeclamptic patients compared with normal controls (9). Noteworthy, C1q depositions were found in the endothelial cells of placental blood vessels and the degree of C1q deposition was significantly higher in controls and early-onset preeclampsia group (EOP; <34 weeks) compared to the late-onset preeclampsia group (LOP; ≥34 weeks) (9). Also C4bp, an inhibitor of the classical pathway was found in 80% of the controls in the syncytiotrophoblast whereas 42% of the preeclamptic placentas had some C4bp deposition in the syncytiotrophoblast layer (9). In early-onset preeclampsia typically more severe placental dysfunction is observed (9). On the other hand, Buurma et al. did observe C1q deposition at the syncytiotrophoblast and in intravillous endothelial cells in preeclamptic and control placentas (11). In both case and control groups, C1q was never entirely negative (11). Comparable results were found regarding to the intensity and distribution of C1q (11). To summarize, the results of C1q deposition varies, this could be the case because studies focused on different parts of the placenta, for example the fetal part or the vasculature of the placenta. Also, a distinction between early-onset and late-onset preeclampsia could explain the varying results. However, these results encourage more research to understand the role of C1q in the pathophysiology of preeclampsia.

According to Kim et al. there is a correlation between C4d staining at the syncytiotrophoblast layer and maternal vascular underperfusion (15). C4d immunoreactivity is significantly more frequent in cases of preterm (<37 weeks) preeclampsia with FGR than in preterm preeclampsia without FGR (15). Also, Buurma et al. found significantly more C4d in preeclamptic placentas compared to healthy controls, and found associations between a diffuse deposition of placental C4d in preeclamptic women and significantly lower gestational ages at delivery (11). According to Buurma et al. the relation of placental C4d and lower gestational age at birth implies that the degree of complement dysregulation is related with the severity of preeclampsia (11). Remarkable, Kim et al. found no association between C4d and the severity of the disease in preeclampsia cases with and without FGR (15). It is possible that C4d immunoreactivity is related with biological challenges such as oxidative stress and endoplasmic reticulum stress in placental cells (15). This could be the consequence of the more severe forms of placental pathology resulting from shallow placentation and resulting in ischemia-reperfusion injury (11, 15). C4d deposition may occur by activation of both the lectin and classical pathway. It is suggested that C4d deposits are the result of classical pathway activation, because C4d co-localized with C1q (11, 33). Also, significantly higher C4d deposition was observed in placentas after preeclampsia compared to placentas after uncomplicated pregnancies, both in pregnancies after oocyte donations, and autologous pregnancies (16).

#### Classical Pathway—Blood

Different studies showed altered complement activation of the classical pathway, C1q, and C4, in peripheral blood in preeclampsia (12–14, 17, 18). Significantly higher circulating levels of C4d, a split product of C4, were observed in women with preeclampsia compared to women with healthy pregnancies (17, 18). Enhanced levels of C4d could be the result of activation of the classical or lectin pathway (17). On the other hand, studies also showed significantly lower circulating levels of C4 in women with preeclampsia compared to normotensive controls (13, 14). Agostinis et al. presented lower levels of C1q in preeclamptic women compared to healthy controls, and also observed a significant decrease in C4, suggesting an ongoing activation of the classical pathway (12). They investigated if these complement factors could be used as early predictive markers of preeclampsia. Quantification of complement factors between weeks 11 and 13 of gestation did not show significant differences between the groups (12). Although C1q and C4 cannot be used as early predictive markers of preeclampsia, it is shown that preeclampsia is associated with altered complement activation (12). It is possible that reduction of circulating levels of C4 as well as C1q are signals of consumption of complement factors due to systemic activation of the immune response (14). Consumption of complement factors is supported by increased formation of split products, like increased deposition and levels of C4d in preeclamptic placentas and circulation compared to controls (14, 17). Another possible explanation is that C1q is removed by binding to cells undergoing apoptosis (12). C1q binds to apoptotic cells and is recognized by phagocytes (12).

### Lectin Pathway

#### Lectin Pathway—Placenta

Focusing on the lectin pathway, ficolins (H, L) were differentially expressed in plasma from preeclamptic pregnancies, compared to healthy controls (19). Ficolins were found in low concentrations in plasma but at high concentrations in the placenta, especially in syncytiotrophoblast undergoing apoptosis when compared with normal placentas (19). Increased trophoblastic apoptosis stimulates placental and systemic innate immunity through ficolin binding (19). The innate immune response can activate the lectin-complement pathway to remove apoptotic cells (19). Another activator of the lectin pathway, MBL, was absent in all placentas (11).

#### Lectin Pathway—Blood

Different study groups measured levels in the circulation of components of the lectin pathway during preeclampsia. When studying MBL, women with preeclampsia have higher median maternal plasma MBL concentrations than controls, and high maternal plasma MBL concentrations were correlated with preeclampsia (20). Also, a larger part of women with preeclampsia have a high MBL concentration (>1898.9 ng/ml) compared to controls, as well as a larger part of controls have low/deficient (0–143.7 ng/ml) plasma MBL concentrations than women with preeclampsia (20). Placental underperfusion was noticed more frequent in patients with high plasma MBL concentration, both in normal pregnant women and women with preeclampsia (20). The exact role of the lectin pathway in preeclampsia is not clear, but the high concentrations in the maternal plasma reflect exaggerated intravascular inflammation, and activation of the immune system in preeclampsia (20).

To determine the functional activity of MBL, Csuka et al. studied the functional complex MBL-MASP2 (mannose-binding lectin-associated serine protease 2) (21). MBL-MASP2 was elevated in healthy pregnant women compared to non-pregnant women (21). However, patients with preeclampsia have comparable circulating levels of MBL-MASP2 as controls. This demonstrates an activation of the lectin pathway during pregnancy, but no further activation during preeclampsia was demonstrated. So, in contrast to Than et al. and Csuka et al. found no relation between the activity of the lectin pathway and pathologic complement activation observed in preeclampsia, suggesting a minor role of the lectin pathway in the development of preeclampsia (20, 21). However, studying polymorphisms in the MBL2 gene, fetal MBL2 haplotypes together with viral exposure are linked to adverse pregnancy outcomes (34).

Ficolins activate the complement system through the lectin pathway by association with effector MASPs (18). However, there was no association between ficolin levels and complement activation products (18). This suggests that the lectin pathway, activated by ficolins, does not play an essential role in complement activation during a healthy pregnancy, and preeclampsia (18). Nevertheless, circulating levels of ficolin-2 (L-ficolin) are decreased in the third trimester of normal pregnancy compared to non-pregnant women (18). There is a further significant reduction in plasma ficolin-2 concentrations in preeclampsia compared to healthy non-pregnant and pregnant women, due to its consumption or primary deficiency of ficolin-2 (18). Women with preeclampsia showed an association between plasma ficolin-2 concentrations and placental growth factor (PIGF) concentrations and an inverse association with serum levels of soluble fms-like tyrosine kinase-1 (sFlt-1) (18). The decrease in ficolin-2 may therefore contribute to the development of endothelial dysfunction and the maternal features preeclampsia (18). Trophoblast apoptosis is normal during pregnancy, but increased in preeclampsia, sometimes dominated by necrosis (18). The underlying mechanism is the ability of ficolins to function as direct opsonins and mediate the clearance of among others apoptotic and necrotic cells through phagocytosis (18). Due to consumption or primary deficiency of ficolin-2 in plasma, it might influence the clearance of apoptotic, and necrotic placental material, released into the maternal circulation, leading to the maternal syndrome of the disease (18).

### Alternative Pathway

#### Alternative Pathway—Placenta

Tedesco et al. were one of the first who found C3 deposition in preeclamptic placentas at the decidua of the basal plate and in the stroma and basal membranes of the villi (22). Additional studies (10, 23, 24) also observed an increase in C3 deposition after immunostaining human placentas of preeclamptic pregnancies compared to controls; however, Lokki et al. did not find a difference (9). In contrast to the other studies, Lokki et al. differentiated between early-onset and late-onset preeclampsia, which might explain the conflicting results (9). Besides that, it is also important to mention that some studies focused on the maternal part of the placenta and others only on the fetal part (9, 10, 22–24).

#### Alternative Pathway—Blood

Complement pathway activation fragment Bb functions as a marker of alternative complement activation, mainly in the circulation (26). In maternal plasma after more than 24 weeks (blood drawn at 32.8 ± 4.4 weeks) of gestation, Bb levels are higher in women with preeclampsia compared to controls (26). Bb levels were significantly higher (nearly 2.5 times) in umbilical venous cord plasma of preeclamptic subjects compared to controls. In addition, there was a significant relation between preeclamptic maternal and umbilical cord plasma Bb levels (26). This may suggest an association between the alternative complement pathway and preeclampsia (26). In contrast to C1q and C4, fragment Bb can also be used in early pregnancy as a predictor of preeclampsia (27). In a normal population, women with elevated Bb (>90th percentile) before 20 weeks of gestation are three times more presumably to develop preeclampsia than women with lower levels (27). This also suggests an association between fragment Bb and preeclampsia (27). Levels of C3 were also measured in the circulation of women with preeclampsia, but these findings were comparable with control groups (14, 25).

### Anaphylatoxins

#### Anaphylatoxins—Placenta

Anaphylatoxin C3a circulates in maternal blood and its action is elicited via the C3a receptor (C3aR) (28). Lim et al. found a decrease in C3aR mRNA expression in placental tissue of severe early-onset preeclamptic women compared to preterm control pregnancies, not affected by preeclampsia (28). Additionally, C3aR protein expression was significantly decreased in women with preeclampsia compared to controls (28, 29). Decreased C3aR might indicate dysregulation of the complement system, inciting inflammation. Whereby immune complexes and necrotic cells are not appropriate lysed in preeclamptic placentas (28) Lim et al. did not find any differences in C5a receptor (C5aR) in contrast to Ma et al. (28, 31) They detected an elevated C5aR expression at trophoblast cells of preeclamptic placentas and suggested that the placental C5a/C5aR pathway supported to the development of preeclampsia by regulating placental trophoblasts dysfunction (31). C5a stimulated trophoblasts toward an anti-angiogenic phenotype by the increased expression of sFlt-1 and a decrease in PIGF (31). A lower C5aR protein expression was found in a larger study than Ma et al. and only focused on severe preeclampsia compared to controls (29). This lower expression might indicate an independent factor in the pathogenesis of severe preeclampsia (29).

#### Anaphylatoxins—Blood

Anaphylatoxins C3a and C5a are often measured and altered in women with preeclampsia compared to healthy pregnant women. Although some studies did not find differences between cases and controls (12, 30) other studies (17, 18, 25) did demonstrate higher circulating levels of C3a in women with preeclampsia compared to controls. After differentiating between early- and late-onset preeclampsia, C3a levels showed a more pronounced elevation in the early than in the late onset preeclampsia group (25). This suggests a different pathophysiology of the two forms of preeclampsia and possibly a more homogenous pathogenesis of the early onset form compared to the late onset form (25). Also, Ye et al. found higher C3a and C5a levels in women with severe preeclampsia than in healthy pregnant women (29). They also showed a positive correlation between C3a and C5a levels and a maternal autoantibody AT1-AA, a potential contributor to the pathogenesis of preeclampsia (29).

### Terminal Pathway

#### Terminal Pathway—Placenta

Already in 1990 higher deposits of the membrane-attack complex, C5b-9, were found in preeclamptic placentas compared to normal placentas (22). Deposits of the terminal complex, C9, were found in the basal membranes of the syncytiotrophoblast and in villous stroma, but these deposits were comparable between preeclampsia (early- and/or late-onset) and control placentas (9). Other studies found in chorionic villi of preeclamptic placentas higher C9 deposition compared to control normal placentas (10, 32). More C9 deposition was found within the fibrinoid parts. Besides that, these higher C9 depositions were more present in placentas from severe forms of preeclampsia than placentas from the mild form (10). These differences could be explained by comparing subgroups with differences in defining the subgroups. The study in question defined severe preeclampsia as >2 g/l proteinuria and mild preeclampsia <2 g/l (10). Based on the weeks of gestation at diagnosis, early-onset preeclampsia was indicated <34 weeks and late-onset >34 weeks of gestation (9). These results suggest a link between preeclampsia and the activation of the complement system (22). The terminal complement complex could be harmful to placental tissue, through a direct lytic effect on cells or by promoting the release of inflammatory products, which can lead to placental dysfunction (22).

#### Terminal Pathway—Blood

The amount of terminal complex formation in preeclamptic women compared to healthy pregnant women differs between studies. Some studies did not find differences between preeclamptic women and controls (12, 30), while several others did (17, 18, 25). Lynch et al. collected blood of a cohort of pregnant women between 10–15 weeks of gestation (30). In this cohort comparable levels of sC5b-9 were found between women who developed preeclampsia and women with a normotensive pregnancy (30). Other studies included women at the time of diagnoses of preeclampsia and mostly found higher levels of sC5b-9 (17, 18, 25). Evidence have shown that preeclamptic women with FGR have significantly higher plasma sC5b-9 levels than those without FGR (17). Besides that, there is a correlation between C3 activation and enhanced terminal complex formation in women with preeclampsia, compared to controls (17).

## Complement Activation in Preeclamptic Animal Models

Next to human studies on the complement system and placental dysfunction, there are studies in mice and rats which investigated the contribution of the complement system in preeclampsia (Table 2). Different mouse or rat models have been used to study placental dysfunction or preeclampsia (Table 3). The animal models are used to mimic human like preeclampsia characteristics and show a strong link with the complement system.

**Table 2:** Overview of complement activation in preeclamptic animal models compared to controls.

**Complement pathway**	**Complement factor**	**Results**	**Animal model**
Classical	C1q	- Increased fetal resorption, reduced fetal weight and impaired development of implantation sites (35) - Abnormal placentation (reduced blood flow and increased oxidative stress) and onset of PE in mice (hypertension, albuminuria, endotheliosis, decreased VEGF and increased levels of sFlt-1 (28)	- C1q deficient compared to wild-type C57BL/6 control mice (35) - C1q deficient compared to wild-type C57BL/6 control mice (28)
Lectin	C4	- Higher deposition on trophoblast and increased rate of fetal resorption when injected with aPL (33)	- C1q and factor D deficient compared to wild-type C57BL/6 mice. Injected with aPL or control human IgG (33)
	MBL-A	- Localized at implantation sites, followed by higher C4, C3, and C9 deposition. Absence of C1r in both groups (36) - Lower C3 and C9 deposition and less fetal resorption in MBL-A deficient mice (36)	- Abortion-prone CBA/J X DBA/2 compared to non-abortion-prone control mice CBA/J X BALB/c (36) - MBL-A^−/−^ compared to MBL-A^+/+^ mice (36)
Alternative	C3	- Increased deposition around the vasculature at E7.5 (37) - Increased C3 together with C9 deposition in the TGC at E10.5 (37) - AT_1_-AA mediated AT_1_-receptor activation contributed to increased C3 deposition in the trophoblast and endothelial cells (24)	- BPH/5 compared to C57BL/6 control mice (37) - C57BL/6J mice; PE-IgG and NT-IgG (24)
Anaphylatoxins	C3a	- Signaling via C3aR is involved in hypertension, proteinuria, sFlt-1, small placental sizes, impaired angiogenesis and IUGR (24)	- C57BL/6J mice; PE-IgG compared to NT-IgG (24)
	C5a	- Increased C5a levels, related to a deficiency of VEGF and increased levels of sFlt-1 (38)	- Abortion-prone mice CBA/J X DBA/2 compared to non-abortion-prone control mice CBA/J X BALB/c (38)

*aPL, antiphospholipid antibodies; AT_1_-AA, angiotensin II type 1 receptor agonistic autoantibody; BPH, blood pressure high; IUGR, intrauterine growth restriction; MBL, mannose-binding lectin; NT, normo-tensive; PE, preeclampsia; s-Flt-1, soluble fms-like tyrosine kinase-1; TGC, trophoblast giant cell; VEGF, vascular endothelial growth factor*.

**Table 3:** Preeclamptic animal models.

**Animal models**	**Features**
Abortion-prone CBA/J X DBA/2 mice	High fetal resorption, fetal growth restriction and defective vascularization and impaired development of the placenta. Features of human preeclampsia: proteinuria and glomerular endotheliosis, but no hypertension (36, 38, 39)
Blood pressure high (BPH/5) mice	Maternal features of preeclampsia and development of placentopathies: shallow trophoblast invasion and inadequate spiral artery remodeling (37, 40)
C1q^−/−^ mice	Deficient in C1q (classical pathway) (28, 35)
C1q and factor D deficient mice	Deficient in C1q (classical pathway) and factor D (alternative pathway) (33)
MBL-A^−/−^ mice	Deficient in MBL-A (lectin pathway) (36)
Reduced utero-placental perfusion pressure (RUPP) rats	Placental ischemia is induced, resulting in reduction of uteroplacental blood flow and hypertension (41–43)

### Animal—Classical Pathway

Agostinis and colleagues examined the contribution the classical pathway, via C1q, and pregnancy outcomes by analyzing implantation sites from C1q^−/−^ mice and controls (35). In C1q-deficient mice, a higher fetal resorption frequency, decreased fetal weight were observed at day 15 of pregnancy (35). Histopathologic analysis of implantation sites from C1q^−/−^ mice showed signs of impaired placental development as compared with controls, like diminished labyrinth development, decreased vascular remodeling, and marked edema in the interstitium of decidual stroma (35). A further study by Singh et al. demonstrated also an association between the absence of C1q, abnormal placentation, and onset of preeclampsia in mice, characterized by hypertension, albuminuria, endotheliosis, decreased vascular endothelial growth factor (VEGF), and elevated levels of sFlt-1 (44).

Further research on the role of the classical pathway in inducing fetal loss was reported by studying a risk factor of developing preeclampsia (33). The complement system plays a pivotal role in the antiphospholipid syndrome, but the exact pathways are unknown. Therefore, pregnant control mice and mice deficient in C1q and factor D were injected with antiphospholipid antibodies (aPL) or control human IgG (33). In the placentas of mice treated with aPL, increased C4 deposition was observed, compared with placentas from normal human IgG treated mice (33). C4 deposition was observed on trophoblast giant cells, which are essential for implantation and invasion of the conceptus into the uterus (33). There was a correlation between C4 deposits and a higher fetal absorption rate (33). Furthermore, mice deficient in C1q and factor D were protected from aPL-induced fetal injury and no C4 deposition was detected (33).

### Animal—Lectin Pathway

The abortion-prone mouse mating combination of CBA/J x DBA/2 is characterized by high fetal resorption and fetal growth restriction (38). This model has been used to understand the role of the complement system in recurrent pregnancy loss and fetal growth restriction, but also shows similarities with preeclampsia (38). This model demonstrates human preeclamptic features, like elevated antiangiogenic factors, placental dysfunction, and proteinuria, but no hypertension. In this mice model, complement was activated primarily through the lectin pathway (36). While only one form of MBL is present in humans, two forms of MBL are found in mice, MBL-A and MBL-C (45). MBL-A was found at the implantation sites of these models. After inhibition of MBL-A activity, MBL-A binding to the decidua was inhibited, together with C3, and C9 deposition, and associated with prevention of pregnancy loss (36, 38).

### Animal—Complement Factor 3

Researchers studying complement factor 3, found increased expression of C3 in vessels and placentas of preeclamptic mice and rat models compared with controls, as seen in Tables 2, 3. Preeclamptic animal models showed symptoms like hypertension and proteinuria (14, 36, 37). Abnormal decidual vasculature with excessive complement deposition was observed around the vasculature in pregnant blood pressure high (BPH)/5 mice, which is a preeclampsia mouse model (37). There was increased expression of C3 around the decidual vasculature of E7.5 implantation sites and in the trophoblast giant cell layer of E10.5 placentas (37). Also, C9 was significantly increased in the trophoblast giant cell layer compared to controls, suggesting activation of the final step of the complement cascade and lysis of the opponent (37). They propose that aberrant maternal (decidual) angiogenesis triggers complement activation before placentation, leading to shallow endovascular trophoblast invasion into the maternal spiral arteries, the first stage of preeclampsia (37).

Wang et al. studied the contribution of AT_1_-AA to C3 elevation in placentas, by injecting IgG from normotensive pregnant women or IgG from women with preeclampsia into pregnant C57 mice (24). Immunohistochemical analysis revealed that C3 was predominantly deposited in the placentas of mice with injected IgG from preeclamptic women. Therefore, IgG from preeclamptic women induces deposition of C3 in placentas of pregnant mice via activation of the AT_1_-receptor (24).

### Animal—Anaphylatoxins (C3a, C5a)

Wang et al. also examined C3aR expression using immunohistochemistry (24). The C3a receptors were highly expressed in trophoblast cells of mouse placentas. After blocking C3aR, autoantibody-induced (AT_1_-AA) hypertension and proteinuria in pregnant mice significantly improved (24). Also, alterations in placenta histology and decreases in placental and fetal weight were prevented, and serum levels of sFlt-1 were reduced (24). This implies a role for C3aR signaling in autoantibody-induced abnormal placentas, FGR and impaired placenta angiogenesis related to preeclampsia (24).

Excessive C5a generation is associated with impaired angiogenesis using the abortion-prone CBA/J x DBA/2 mouse mating combination, as mentioned before (38). The increased C5a levels were related to a deficiency of VEGF and increased levels of sFlt-1 (38). This suggests a relation between increased circulating levels of anaphylatoxins and the pathophysiology of preeclampsia.

## Inhibition of the Complement System in Preeclamptic Animal Models

Therapeutic studies in animals prove the effect of inhibition of the complement system in improving preeclampsia (Table 4). Lillegard et al. used the reduced utero-placental perfusion pressure (RUPP) rat model to induce hypertension in the third trimester and studied the effect of decreasing complement activation with a soluble recombinant form of an endogenous complement regulator, human complement receptor 1 (sCR1) (41). Compared to controls, preeclampsia prone rats showed decreased circulating C3 and increased C3a, indicating that complement activation has occurred. sCR1 inhibited complement activation by reducing the production of C3a and reducing the elevation of the mean arterial pressure (MAP) (41). VEGF was also decreased in RUPP rats compared to controls, but VEGF was not affected by sCR1 treatment (41). So, sCR1 did not reverse RUPP-induced FGR. This suggests that the role of complement in placental ischemia-induced hypertension was independent of VEGF (41).

**Table 4:** Overview of complement inhibition in preeclamptic animal models compared to controls.

**Complement pathway**	**Complement inhibitor**	**Results**	**Animal model**
Classical	sCR1	- Reduced C3a levels and reduced elevated MAP (41)	RUPP compared to sham control rats (41)
Lectin	CR2-FH	- Comparable placental weights (4)	BPH/5 compared to C57BL/6J control mice (40)
All pathways	CR2-Crry	- Increased placental weights (40) - Inhibition of oxidative stress, proteinuria and placental dysfunction (39)	BPH/5 compared to C57BL/6J control mice (40) Abortion-prone CBA/J X DBA/2 compared to non-abortion-prone control mice CBA/J X BALB/c (39)
Anapyhlatoxins	C3a and/or C5a antagonist	- Reduced increase in MAP, without altering the decreased fetal weight or VEGF (42) - C5a antagonists attenuated endothelial dysfunction (42)	RUPP compared to sham control rats (41, 42)
	Atrasentan	- Improvement of hypertension (43) - Comparable fetal and placental weights (43)	RUPP compared to sham control rats (43)

*BPH, blood pressure high; MAP, mean arterial pressure; RUPP, reduced utero-placental perfusion pressure; VEGF, vascular endothelial growth factor*.

Lillegard et al. further investigated the RUPP rat model (42). They treated rats with C3aR or C5aR antagonists and found a significant inhibition of the RUPP-induced increase in MAP compared to controls (42). Treatment of rats with a combination of both antagonists did not result in a better MAP than treatment with one antagonist alone (42). Again, VEGF did not change after treatment with C3aR and/or C5aR antagonists (42). In addition, only C5aR antagonists attenuated endothelial dysfunction followed by placental ischemia in the RUPP model rats compared to controls (42). The mechanism by which anaphylatoxins C3a and C5a contribute to placental ischemia seems to be different (42).

In addition to the previous study, they hypothesized that complement activation following placental ischemia leads to activation of the endothelin pathway which triggers hypertension and FGR (43). However, they suggested a complex interaction of the endothelin and complement system in normal pregnancies (43). After treatment with endothelin A receptor antagonist atrasentan hypertension significantly improved, but fetal and placental weight did not differ in the RUPP surgery groups (43). Concerning the complement factors, circulating C3a was increased in the RUPP surgery groups compared to controls, but C3a was unaffected in the RUPP surgery groups after treatment (43). Surprisingly, a significantly increased placental C3 deposition was only observed in the control group after treatment (43). This is explained by a significant downregulation of normal complement regulators (CD55/Crry) *in vivo* placentas by the endothelin antagonist (43).

Gelber et al. also studied the BPH/5 hypertensive mice characterized by fetal loss and growth restriction in association with abnormal placentation and defects in maternal decidual arteries (40). They blocked all pathways of complement activation with CR2-Crry and selectively inhibited the alternative pathway with CR2-FH. Local complement inhibition was related with increased weight of the placenta in the CR2-Crry treated mice, but not in those treated with CR2-FH compared to controls (40). Also, other researchers blocked complement activation at the maternal-fetal interface by administering a single dose of CR2-Crry in preeclamptic mice. Inhibition prevented among others oxidative stress and placental dysfunction in these mice compared to controls (39).

## Pharmaceutical Interventions as Prevention or Treatment of Preeclampsia

Nowadays, treatment of preeclampsia is achieved by delivery of the placenta and therefore of the fetus. Therapeutic options are mostly symptomatic and not linked to underlying causes. Currently, Low-dose Aspirin (LDA) + low-molecular-weight heparin (LMWH) are the most common preventive therapies for preeclampsia, but several studies have been performed to investigate other potential therapies to postpone delivery and protect mother and fetus (Table 5). Pharmaceutical interventions as treatment or prevention for preeclampsia will be discussed, the underlying mechanism and their link with the complement system.

**Table 5:** Overview of pharmaceutical interventions and their possible mechanism improving preeclampsia.

**Study**	**Study design**	**Drug**	**Possible mechanism improving preeclampsia**
Bujold et al. (46) Roberge et al. (47)	Meta-analysis	Aspirin	Improves trophoblastic invasion of the uterine spiral arteries. Down-regulates the placental expression of C3 and of complement factor B.
Rodger et al. (48) Roberge et al. (49) Wang et al. (50)	Meta-analysis	Heparin	Improves maternal vasculature, endothelial function, increases placental growth factor and inhibits of C5a.
Seo et al. (51)	Retrospective cohort	Hydroxy-chloroquine	Diverse molecular pathways, incl. antioxidant, anti-inflammatory, immunomodulatory and antithrombotic. Impair complement-dependent antigen-antibody reactions.
Lefkou et al. (6)	Case-control	Pravastatin	Diminishes inflammation, increases placental blood flow, and reverses angiogenic and redox imbalances. Inhibits complement activation by induction of DAF expression.
Sones et al. (52) Reijnders et al. (53)	Case-control (mice)	Celecoxib	Increases uterine vascular permeability and angiogenesis. Does not significantly decrease complement (C3, complement factor B) expression in mice.
Burwick et al. (54)	Case-report	Eculizumab	Inhibits C5, reduces C5a, and C5b-9 generation.

### Drugs Investigated as Prevention for Preeclampsia

#### Aspirin

Acetylsalicylic acid has both anti-inflammatory and antipyretic effects. In a lower dose it inhibits platelet aggregation via prostaglandin thromboxane A_2_. Several studies have investigated the role of aspirin in preventing preeclampsia, with conflicting results. A possible mechanism explaining that aspirin reduces the risk of preeclampsia, is the improvement in trophoblastic invasion of the uterine spiral arteries (46). Down-regulation of placental expressions of complement factor 3 and factor B is also suggested by LDA-treatment (55). A recent meta-analysis examined the effect of aspirin in the prevention of preterm and term preeclampsia in relation to gestational age at onset of treatment and the dose of aspirin (47). They included 16 studies, also the large Combined Multimarker Screening and Randomized Patient Treatment with Aspirin for Evidence-Based Preeclampsia Prevention (ASPRE) trial, for a total of 18,907 participants between 1985 and 2017 (47, 56). Their conclusion, aspirin starting at ≤16 weeks of gestation and at a dose of ≥100 mg/day decreases the risk of preterm preeclampsia by approximately 70% (47). Aspirin did not reduce risk of term preeclampsia (47). These findings were also suggested earlier in a meta-analysis by Bujold et al. (46).

#### Heparin

Last years, clinical trials have studied the prevention of preeclampsia by LMWH (50). The pathways by which LMWH could prevent preeclampsia are not clear. Experiments have demonstrated that LMWH exerts positive actions directly on the maternal vasculature, endothelial function, increasing circulating maternal levels of PIGF and inhibiting complement activation (C5a) (57, 58). Overall, it is not clear if LMWH is capable of suppressing one or various pathways that could contribute to the development of preeclampsia (57). It might be that just a subgroup of women at risk for early-onset preeclampsia-associated placental and maternal cardiovascular dysfunction may profit from LMWH, which may explain the conflicting trial results (57, 58). Heparin has been found to influence complement pathways in both *in vitro* and *in vivo* studies. It inhibits the effects of complement pathways on the trophoblast; however especially in mouse models of antiphospholipid syndrome (59).

The conclusions of the studies analyzing the use of a LMWH as preventive therapy for preeclampsia are conflicting. Rodger et al. performed an individual patient data meta-analysis including 963 eligible women from eight trials (48). They noted significant heterogeneity between single center and multicenter trials and suggests that administration of LMWH does not have an effect on the prevention of placenta-mediated pregnancy complications, including preeclampsia (48). However, they also suggest that additional large multicenter clinical studies are needed (48). Furthermore, Wang et al. and Roberge et al. performed a meta-analysis and compared LMWH plus LDA with LDA only (49, 50). In addition, Wang et al. also included two studies comparing a LMWH group with a no LDA group and Roberge et al. also included unfractionated heparin. The gestational ages of the enrolled women were all within 16 weeks (50). Wang et al. and Roberge et al. found in, respectively seven studies (1,035 participants) and eight studies (885 participants) that LMWH effectively reduces the risk of preeclampsia in pregnant women with a history of early onset or severe preeclampsia (49, 50). Prophylactic use of LMWH prolongs gestational length and increases neonatal weight (50). Both studies propose that there is a need for more larger multicenter research, also including more screening indicators, for example uterine artery Doppler ultrasound and serum markers (49, 50). This will create more accurate screening of high-risk groups of preeclampsia and will gain more accurate and reliable evaluations of the prevention efficacy of LMWH in these group of pregnant women (49, 50).

#### Hydroxychloroquine

Hydroxychloroquine (HCQ), an antimalarial drug, is used in the treatment of systemic lupus erythematosus (SLE) (51). It is effective in preventing flares, achieving remission, and reducing overall mortality (51). HCQ has several effects, including antioxidant, anti-inflammatory, immunomodulatory, and antithrombotic (51). It acts by impediment to lysosomal function, interfering with antigen processing in macrophages and other antigen-presenting cells, inhibition of Toll-like receptors-associated mechanisms thereby diminishing the production of proinflammatory cytokines and also impair complement- dependent antigen–antibody reactions (51, 60). Women with SLE have a higher risk of developing preeclampsia than women without SLE. This drug has been used in pregnant women with SLE and it is also recommended to continue during pregnancy for maintaining remission or to treat a flare (51). In 2019, Seo et al. compared pregnancy outcomes, including preeclampsia, in a retrospective study of SLE patients treated during pregnancy with hydroxychloroquine (51). The incidence of preeclampsia was significantly lower in the HCQ treatment group than in the HCQ non-treatment group (51). Although the use of aspirin during pregnancy was significantly higher in the HCQ treatment group than in the HCQ non-treatment group, after correcting for aspirin use, HCQ treatment group showed a lower incidence of preeclampsia compared to the HCQ non-treatment group (51). Despite the assumption that HCQ could inhibit the complement system, levels of circulating C4 and C3 did not differ between the groups. So its main effect will not exert via the complement system (51).

### Drugs Investigated as Treatment for Preeclampsia

#### Pravastatin

Recently, Lefkou et al. assessed pregnancies outcomes in women with antiphospholipid syndrome (APS) that developed preeclampsia and/or FGR despit e the use of LDA+LMWH, with additional pravastatin (HMG-CoA reductase inhibitor) (6). At the time of the onset of placental insufficiency, pravastatin was administered (6). Twenty-one pregnant women with APS developed preeclampsia and/or FGR during treatment with LDA + LMWH (6). The control group (*n* = 10) received only LDA+LMWH and 11 pregnant women received pravastatin in addition to LDA+LMWH at the onset of preeclampsia and/or FGR (6). The trial was unblinded. All control patients delivered preterm by emergency C-section because of fetal and/or maternal health concerns and three perinatal deaths were observed. Pravastatin prolonged pregnancies significantly after the onset of preeclampsia compared with those who did not receive pravastatin. All patients with pravastatin delivered after 34 weeks with good neonatal outcomes and showed increased placental blood flow and improvements in preeclampsia features (6). Women who received pravastatin delivered close to term (median, 36 weeks; IQR 35–36), which is beneficial for the development of the fetus (6). This study analyzed the use of pravastatin in pregnant women with APS at an increased risk of adverse pregnancy outcomes (6). The mechanism of action of pravastatin involves the complement system, by preventing cervical remodeling and myometrial contractions (61). In mice studies pravastatin inhibited complement activation (C5a) in the cervix by increasing the production and expression of complement inhibitor DAF (61).

#### Celecoxib

Celecoxib is a non-steroidal anti-inflammatory drug, inhibits cyclooxygenase−2 (COX-2), and the production of prostaglandins (PGE) (52). Implantation is important for pregnancy outcome (52, 53). This is characterized by increased uterine vascular permeability and angiogenesis and is under the influence of among others COX-2 and prostaglandins (52, 53). Sones et al. determined the effect of celecoxib in BPH/5 mice, administered before placentation (52). They studied if angiogenic factor imbalance and complement dysregulation improved after therapy (52). They compared celecoxib-treated BPH/5 mice with vehicle-treated BPH/5 and C57 mice controls. After a single dose of a COX-2 inhibitor during pregnancy (E6.5) results showed significant reduction in COX2 protein an PGE_2_ levels, improved fetal growth, normalized placental weight, and diminished late gestational hypertension in BPH/5 mice (52). Further study also showed that celecoxib restores angiogenic factor expression at the maternal-fetal interface in the BPH/5 mouse model (53). Furthermore, placental complement expression after celecoxib treatment showed an increase in C3 and complement factor B mRNA in E10.5 placentas from control BPH/5 pregnancies as compared with C57 vehicle-treated controls (53). However, there were no differences in placental C3 and complement factor B mRNA expressions between celecoxib-treated and vehicle-treated BPH/5 pregnancies (53). These studies provided evidence that decidual overexpression of COX-2 may trigger adverse pregnancy outcomes seen in preeclampsia, although celecoxib treatment is not sufficient to prevent aberrant complement expression in BPH/5 placentas (52, 53).

#### Eculizumab

Eculizumab is a monoclonal antibody inhibitor of complement factor 5 and decreases the generation of complement components C5a and C5b-9 and inhibit their effects (54). A case report described a patient presenting with severe preeclampsia/HELLP syndrome at 26 weeks of gestation (54). After treatment with Eculizumab (1,200 mg) maternal and fetal status were reassuring, laboratory parameters (LDH, haptoglobin, AST, ALT, and platelet) improved and pregnancy was prolonged by 17 days (54). Maternal blood pressure fluctuated during treatment, but eventually worsened to 140–160/90–100 mmHg together with other laboratory parameters and delivery was advised (54). However, complement proteins, angiogenic markers or inflammatory cytokines were not measured (54). This report describes the use of Eculizumab supports a possible benefit for the treatment of preeclampsia by C5 inhibition. It also suggests the involvement of the complement system in preeclampsia. Therefore, it may be a treatment option for preeclampsia (54).

#### Conclusion/ Future Perspectives

There is need for an effective therapy to treat or prevent preeclampsia, because of the severe maternal and fetal complications. Based on the discussed literature, there is evidence that the complement system plays an essential role in the pathogenesis of preeclampsia. The results of excessive activation or inadequate regulation of the complement system are increased circulating anaphylatoxins, increased deposition of complement factors in the placenta and consumption of circulating complement factors, leading to placental dysfunction. Current therapy options are predominantly symptomatic and might not intervene with the underlying pathophysiology of preeclampsia. Modulation of the complement system deserves attention, because data with pharmaceutical interventions to investigate the complement system as a potential target to treat preeclampsia are still scarce. In the future, studies are needed to investigate the safety and efficacy of complement modulation to treat or prevent preeclampsia. This can first be done in animal experiments investigating the safety and efficacy of complement modulation in preeclamptic models and thereafter in clinical trials, at which safety of mother and fetus is always paramount.

## Author Contributions

EP built the search strategy, performed the literature study, and wrote the first draft of the manuscript. HG, GD, MD, and MS reviewed and edited the manuscript. JP and SS reviewed and edited the manuscript and supervised the project.

### Conflict of Interest

The authors declare that the research was conducted in the absence of any commercial or financial relationships that could be construed as a potential conflict of interest.

## References

[1] Denny KJ Woodruff TM Taylor SM Callaway LK. Complement in pregnancy: a delicate balance. Am J Reprod Immunol. (2013) 69:3–11. 10.1111/aji.12000 22925193

[2] Tincani A Cavazzana I Ziglioli T Lojacono A De Angelis V Meroni P. Complement activation and pregnancy failure. Clin Rev Allergy Immunol. (2010) 39:153–9. 10.1007/s12016-009-8183-5 19936969

[3] Girardi G. Complement activation, a threat to pregnancy. Semin Immunopathol. (2018) 40:103–11. 10.1007/s00281-017-0645-x 28900713

[4] Robertson SA Green ES Care AS Moldenhauer LM Prins JR Louise Hull M. Therapeutic potential of regulatory T cells in preeclampsia-opportunities and challenges. Front Immunol. (2019) 10:1–18. 10.3389/fimmu.2019.00478 30984163 PMC6448013

[5] Penning M Chua JS Van Kooten C Zandbergen M Buurma A Schutte J. Classical complement pathway activation in the kidneys of women with preeclampsia. Hypertension. (2015) 66:117–25. 10.1161/HYPERTENSIONAHA.115.05484 25941343 PMC4465860

[6] Lefkou E Rousso D Girardi G. Pravastatin improves pregnancy outcomes in obstetric antiphospholipid syndrome refractory to antithrombotic therapy. J Clin Investig. (2016) 126:2933–40. 10.1172/JCI86957 27454295 PMC4966313

[7] Dunkelberger JR Song W-C. Complement and its role in innate and adaptive immune responses. Cell Res. (2010) 20:34. 10.1038/cr.2009.139 20010915

[8] Girardi G Prohászka Z Bulla R Tedesco F Scherjon S. Complement activation in animal and human pregnancies as a model for immunological recognition. Mol Immunol. (2011) 48:1621–30. 10.1016/j.molimm.2011.04.011 21600656

[9] Lokki AI Heikkinen-Eloranta J Jarva H Saisto T Lokki ML Laivuori H. Complement activation and regulation in preeclamptic placenta. Front Immunol. (2014) 5:312. 10.3389/fimmu.2014.00312 25071773 PMC4088925

[10] Sinha D Wellst M Faulkt WP. Immunological studies of human placentae: complement components in pre-eclamptic chorionic villi. Clin. Exp. Immunol. (1984) 56:175–84. 6370518 PMC1535962

[11] Buurma A Cohen D Veraar K Schonkeren D Claas FH Bruijn JA. Preeclampsia is characterized by placental complement dysregulation. Hypertension. (2012) 60:1332–7. 10.1161/HYPERTENSIONAHA.112.194324 23006730

[12] Agostinis C Stampalija T Tannetta D Loganes C Vecchi Brumatti L De Seta F Complement component C1q as potential diagnostic but not predictive marker of preeclampsia. Am J Reprod Immunol. (2016) 76:475–81. 10.1111/aji.12586 27666323

[13] Kestlerová A Feyereisl J Frisová V Měchurová A Šula K Zima T. Immunological and biochemical markers in preeclampsia. J Reprod Immunol. (2012) 96:90–4. 10.1016/j.jri.2012.10.002 23131770

[14] Sarween N Drayson MT Hodson J Knox EM Plant T Day CJ. Humoral immunity in late-onset pre-eclampsia and linkage with angiogenic and inflammatory markers. Am J Reprod Immunol. (2018) 80:e13041. 10.1111/aji.13041 30168226

[15] Kim EN Yoon BH Lee JY Hwang D Kim KC Lee JH. Placental C4d deposition is a feature of defective placentation: observations in cases of preeclampsia and miscarriage. Virchows Arch. (2015) 466:717–25. 10.1007/s00428-015-1759-y 25820373

[16] Lashley LEELO Buurma A Swings GMJS Eikmans M Anholts JDH Bakker JA. Preeclampsia in autologous and oocyte donation pregnancy: is there a different pathophysiology? J Reprod Immunol. (2015) 109:17–23. 10.1016/j.jri.2015.03.004 25863695

[17] Derzsy Z Prohászka Z Rigó J Füst G Molvarec A. Activation of the complement system in normal pregnancy and preeclampsia. Mol Immunol. (2010) 47:1500–6. 10.1016/j.molimm.2010.01.021 20181396

[18] Halmos A Rigó J Szijártó J Füst G Prohászka Z Molvarec A. Circulating ficolin-2 and ficolin-3 in normal pregnancy and pre-eclampsia. Clin Exp Immunol. (2012) 169:49–56. 10.1111/j.1365-2249.2012.04590.x 22670778 PMC3390473

[19] Wang CC Yim KW Poon TCW Choy KW Chu CY Lui WT. Innate immune response by ficolin binding in apoptotic placenta is associated with the clinical syndrome of preeclampsia. Clin Chem. (2007) 53:42–52. 10.1373/clinchem.2007.074401 17202497

[20] Than NG Romero R Erez O Kusanovic JP Tarca AL Edwin SS. A role for mannose-binding lectin, a component of the innate immune system in pre-eclampsia. Am J Reprod Immunol. (2008) 60:333–45. 10.1111/j.1600-0897.2008.00631.x 18727690 PMC2775464

[21] Csuka D Molvarec A Derzsy Z Varga L Füst G Rigó J. Functional analysis of the mannose-binding lectin complement pathway in normal pregnancy and preeclampsia. J Reprod Immunol. (2010) 87:90–6. 10.1016/j.jri.2010.07.004 20952075

[22] Tedesco F Radillo O Candussi G Nazzaro A Mollnes TE Pecorari D. Immunohistochemical detection of terminal complement complex and S protein in normal and pre-eclamptic placentae. Clin Exp Immunol. (1990) 80:236–40. 10.1111/j.1365-2249.1990.tb05240.x 2357851 PMC1535286

[23] Hering L Herse F Verlohren S Park JK Wellner M Qadri F. Trophoblasts reduce the vascular smooth muscle cell proatherogenic response. Hypertension. (2008) 51:554–9. 10.1161/HYPERTENSIONAHA.107.102905 18195163

[24] Wang W Irani RA Zhang Y Ramin SM Blackwell SC Tao L. Autoantibody-mediated complement c3a receptor activation contributes to the pathogenesis of preeclampsia. Hypertension. (2012) 60:712–21. 10.1161/HYPERTENSIONAHA.112.191817 22868393 PMC4131740

[25] Boij R Svensson J Nilsson-Ekdahl K Sandholm K Lindahl TL Palonek E. Biomarkers of coagulation, inflammation, and angiogenesis are independently associated with preeclampsia. Am J Reprod Immunol. (2012) 68:258–70. 10.1111/j.1600-0897.2012.01158.x 22626009

[26] Hoffman MC Rumer KK Kramer A Lynch AM Winn VD. Maternal and fetal alternative complement pathway activation in early severe preeclampsia. Am J Reprod Immunol. (2014) 71:55–60. 10.1111/aji.12162 24128411 PMC4067768

[27] Lynch AM Murphy JR Byers T Gibbs RS Neville MC Giclas PC. Alternative complement pathway activation fragment Bb in early pregnancy as a predictor of preeclampsia. Am J Obstet Gynecol. (2008) 198:385.e1–e9. 10.1016/j.ajog.2007.10.793 18221926 PMC2362503

[28] Lim R Lappas M. Decreased expression of complement 3a receptor (C3aR) in human placentas from severe preeclamptic pregnancies. Eur J Obstet Gynecol Reprod Biol. (2012) 165:194–8. 10.1016/j.ejogrb.2012.08.003 22901903

[29] Ye Y Kong Y Zhang Y. Complement split products C3a/C5a and receptors: are they regulated by circulating angiotensin II type 1 receptor autoantibody in severe preeclampsia? Gynecol Obstet Invest. (2016) 81:28–33. 10.1159/000440651 26485247

[30] Lynch AM Murphy JR Gibbs RS Levine RJ Giclas PC Salmon JE. The interrelationship of complement-activation fragments and angiogenesis-related factors in early pregnancy and their association with pre-eclampsia. BJOG An Int J Obstet Gynaecol. (2010) 117:456–62. 10.1111/j.1471-0528.2009.02473.x 20074261 PMC5267351

[31] Ma Y Kong LR Ge Q Lu YY Hong MN Zhang Y. Complement 5a-mediated trophoblasts dysfunction is involved in the development of pre-eclampsia. J Cell Mol Med. (2018) 22:1034–46. 10.1111/jcmm.13466 29168351 PMC5783881

[32] Banadakoppa M Balakrishnan M Yallampalli C. Upregulation and release of soluble fms-like tyrosine kinase receptor 1 mediated by complement activation in human syncytiotrophoblast cells. Am J Reprod Immunol. (2018) 80:1–11. 10.1111/aji.13033 30099798 PMC6202180

[33] Cohen D Buurma A Goemaere NN Girardi G Le Cessie S Scherjon S. Classical complement activation as a footprint for murine and human antiphospholipid antibody-induced fetal loss. J Pathol. (2011) 225:502–11. 10.1002/path.2893 21688269

[34] Gibson CS MacLennan AH Haan EA Priest K Dekker GA. Fetal MBL2 haplotypes combined with viral exposure are associated with adverse pregnancy outcomes. J Matern Neonatal Med. (2011) 24:847–54. 10.3109/14767058.2010.531324 21171930

[35] Agostinis C Bulla R Tripodo C Gismondi A Stabile H Bossi F. An alternative role of C1q in cell migration and tissue remodeling: contribution to trophoblast invasion and placental development. J Immunol. (2010) 185:4420–9. 10.4049/jimmunol.0903215 20810993

[36] Petitbarat M Durigutto P Macor P Bulla R Palmioli A Bernardi A. Critical role and therapeutic control of the lectin pathway of complement activation in an abortion-prone mouse mating. J Immunol. (2015) 195:5602–7. 10.4049/jimmunol.1501361 26561549

[37] Sones JL Merriam AA Seffens A Brown-Grant DA Butler SD Zhao AM. Angiogenic factor imbalance precedes complement deposition in placentae of the BPH/5 model of preeclampsia. FASEB J. (2018) 32:2574–86. 10.1096/fj.201701008R 29279353 PMC5901389

[38] Girardi G Yarilin D Thurman JM Holers VM Salmon JE. Complement activation induces dysregulation of angiogenic factors and causes fetal rejection and growth restriction. J Exp Med. (2006) 203:2165–75. 10.1084/jem.20061022 16923853 PMC2118387

[39] Qing X Redecha PB Burmeister MA Tomlinson S D'Agati VD Davisson RL. Targeted inhibition of complement activation prevents features of preeclampsia in mice. Kidney Int. (2011) 79:331–9. 10.1038/ki.2010.393 20944547

[40] Gelber SE Brent E Redecha P Perino G Tomlinson S Davisson RL. Prevention of defective placentation and pregnancy loss by blocking innate immune pathways in a syngeneic model of placental insufficiency. J Immunol. (2015) 195:1129–38. 10.4049/jimmunol.1402220 26071558 PMC4506873

[41] Lillegard KE Johnson AC Lojovich SJ Bauer AJ Marsh HC Gilbert JS. Complement activation is critical for placental ischemia-induced hypertension in the rat. Mol Immunol. (2013) 56:91–7. 10.1016/j.molimm.2013.04.009 23685261 PMC3687024

[42] Lillegard KE Loeks-Johnson AC Opacich JW Peterson JM Bauer AJ Elmquist BJ. Differential effects of complement activation products C3a and C5a on cardiovascular function in hypertensive pregnant rats. J Pharmacol Exp Ther. (2014) 351:344–51. 10.1124/jpet.114.218123 25150279 PMC4201271

[43] Regal JF Lund JM Wing CR Root KM McCutcheon L Bemis LT. Interactions between the complement and endothelin systems in normal pregnancy and following placental ischemia. Mol Immunol. (2019) 114:10–8. 10.1016/j.molimm.2019.06.015 31326653 PMC6774867

[44] Singh J Ahmed A Girardi G. Role of complement component C1q in the onset of preeclampsia in mice. Hypertension. (2011) 58:716–24. 10.1161/HYPERTENSIONAHA.111.175919 21859968

[45] De Vries B Walter SJ Peutz-Kootstra CJ Wolfs TGAM Van Heurn LWE Buurman WA. The mannose-binding lectin-pathway is involved in complement activation in the course of renal ischemia-reperfusion injury. Am J Pathol. (2004) 165:1677–88. 10.1016/S0002-9440(10)63424-4 15509537 PMC1618654

[46] Bujold E Roberge S Nicolaides KH. Low-dose aspirin for prevention of adverse outcomes related to abnormal placentation. Prenat Diagn. (2014) 34:642–8. 10.1002/pd.4403 24799357

[47] Roberge S Bujold E Nicolaides KH. Aspirin for the prevention of preterm and term preeclampsia: systematic review and metaanalysis. Am J Obstet Gynecol. (2018) 218:287–93. 10.1016/j.ajog.2017.11.561 29138036

[48] Rodger MA Gris JC de Vries JIP Martinelli I Rey É Schleussner E. Low-molecular-weight heparin and recurrent placenta-mediated pregnancy complications: a meta-analysis of individual patient data from randomised controlled trials. Lancet. (2016) 388:2629–41. 10.1016/S0140-6736(16)31139-4 27720497

[49] Roberge S Demers S Nicolaides KH Bureau M Côté S Bujold E. Prevention of pre-eclampsia by low-molecular-weight heparin in addition to aspirin: a meta-analysis. Ultrasound Obstet Gynecol. (2016) 47:548–53. 10.1002/uog.15789 26481090

[50] Wang X Gao H. Prevention of preeclampsia in high-risk patients with low-molecular-weight heparin: a meta-analysis. J Matern Fetal Neonatal Med. (2018). 10.1080/14767058.2018.1543656. [Epub ahead of print]. 30458652

[51] Seo MR Chae J Kim YM Cha HS Choi SJ Oh S. Hydroxychloroquine treatment during pregnancy in lupus patients is associated with lower risk of preeclampsia. Lupus. (2019) 28:722–30. 10.1177/0961203319843343 30971164

[52] Sones JL Cha J Woods AK Bartos A Heyward CY Lob HE. Decidual Cox2 inhibition improves fetal and maternal outcomes in a preeclampsia-like mouse model. JCI Insight. (2016) 1:1–16. 10.1172/jci.insight.75351 27159542 PMC4855694

[53] Reijnders D Liu C-C Xu X Zhao A Olson K Butler SD. Celecoxib restores angiogenic factor expression at the maternal-fetal interface in the BPH/5 mouse model of preeclampsia. Physiol Genomics. (2018) 50:385–92. 10.1152/physiolgenomics.00115.2017 29521599 PMC6008120

[54] Burwick RM Feinberg BB. Eculizumab for the treatment of preeclampsia/HELLP syndrome. Placenta. (2013) 34:201–3. 10.1016/j.placenta.2012.11.014 23228435

[55] Ducat A Vargas A Doridot L Bagattin A Lerner J Vilotte J-L. Low-dose aspirin protective effects are correlated with deregulation of HNF factor expression in the preeclamptic placentas from mice and humans. Cell Death Discov. (2019) 5:94. 10.1038/s41420-019-0170-x 31098302 PMC6510804

[56] Rolnik DL Wright D Poon LC Aspirin versus placebo in pregnancies at high risk for preterm pre-eclampsia. N Engl J Med. (2017) 377:613–22. 10.1056/NEJMoa1704559 28657417

[57] Wat JM Hawrylyshyn K Baczyk D Greig IR Kingdom JC. Effects of glycol-split low molecular weight heparin on placental, endothelial, and anti-inflammatory pathways relevant to preeclampsia. Biol Reprod. (2018) 99:1082–90. 10.1093/biolre/ioy127 29860275 PMC6297285

[58] McLaughlin K Scholten RR Parker JD Ferrazzi E Kingdom JCP. Low molecular weight heparin for the prevention of severe preeclampsia: where next? Br J Clin Pharmacol. (2018) 84:673–8. 10.1111/bcp.13483 29226532 PMC5867115

[59] Hossain N Schatz F Paidas MJ. Heparin and maternal fetal interface: why should it work to prevent pregnancy complications? Thromb Res. (2009) 124:653–5. 10.1016/j.thromres.2009.08.001 19716166

[60] Mekinian A Costedoat-Chalumeau N Masseau A Tincani A De Caroli S Alijotas-Reig J. Obstetrical APS: is there a place for hydroxychloroquine to improve the pregnancy outcome? Autoimmun Rev. (2015) 14:23–9. 10.1016/j.autrev.2014.08.040 25179813

[61] Gonzalez JM Pedroni SMA Girardi G. Statins prevent cervical remodeling, myometrial contractions and preterm labor through amechanism that involves hemoxygenase-1 and complement inhibition. Mol Hum Reprod. (2014) 20:579–89. 10.1093/molehr/gau019 24623738

